# The clinical utility of bone marker measurements in osteoporosis

**DOI:** 10.1186/1479-5876-11-201

**Published:** 2013-08-29

**Authors:** Gillian Wheater, Mohsen Elshahaly, Stephen P Tuck, Harish K Datta, Jacob M van Laar

**Affiliations:** 1 Department of Biochemistry, The James Cook University Hospital, Middlesbrough TS4 3BW, UK; 2 Institute of Cellular Medicine, Musculoskeletal Research Group, Newcastle University, Newcastle upon Tyne NE1 7RU, UK; 3 Department of Rheumatology, The James Cook University Hospital, Middlesbrough TS4 3BW, UK

**Keywords:** Bone turnover markers, Bone formation, Bone resorption, Osteoporosis, Biological variability

## Abstract

Osteoporosis is characterised by low bone mass and structural deterioration of bone tissue, resulting in increased fragility and susceptibility to fracture. Osteoporotic fractures are a significant cause of morbidity and mortality. Direct medical costs from such fractures in the UK are currently estimated at over two billion pounds per year, resulting in a substantial healthcare burden that is expected to rise exponentially due to increasing life expectancy. Currently bone mineral density is the WHO standard for diagnosis of osteoporosis, but poor sensitivity means that potential fractures will be missed if it is used alone. During the past decade considerable progress has been made in the identification and characterisation of specific biomarkers to aid the management of metabolic bone disease. Technological developments have greatly enhanced assay performance producing reliable, rapid, non-invasive cost effective assays with improved sensitivity and specificity. We now have a greater understanding of the need to regulate pre-analytical sample collection to minimise the effects of biological variation. However, bone turnover markers (BTMs) still have limited clinical utility. It is not routinely recommended to use BTMs to select those at risk of fractures, but baseline measurements of resorption markers are useful before commencement of anti-resorptive treatment and can be checked 3–6 months later to monitor response and adherence to treatment. Similarly, formation markers can be used to monitor bone forming agents. BTMs may also be useful when monitoring patients during treatment holidays and aid in the decision as to when therapy should be recommenced. Recent recommendations by the Bone Marker Standards Working Group propose to standardise research and include a specific marker of bone resorption (CTX) and bone formation (P1NP) in all future studies. It is hoped that improved research in turn will lead to optimised markers for the clinical management of osteoporosis and other bone diseases.

## Introduction

**Figure 1:**
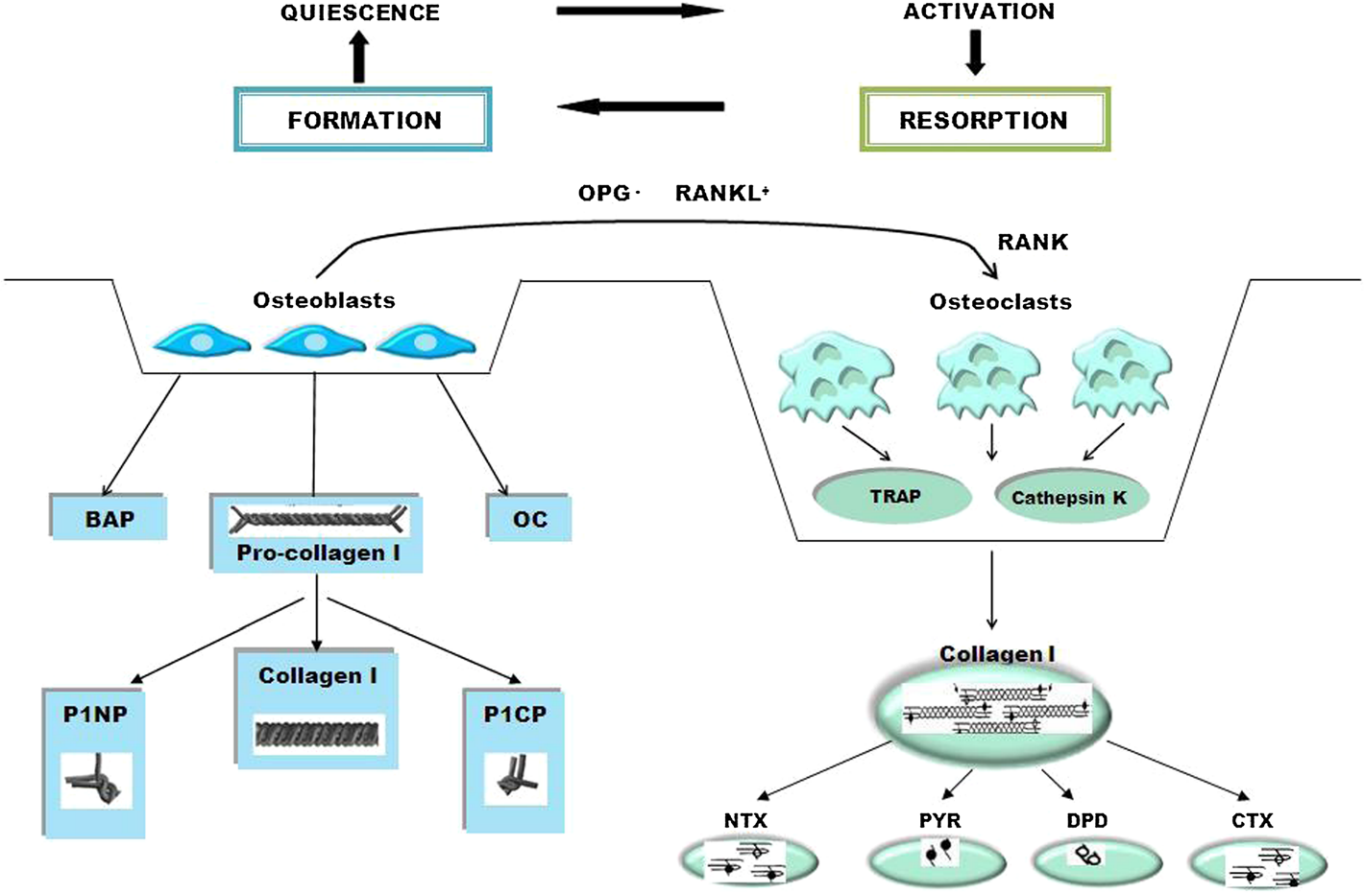
**The bone remodelling cycle.** The bone remodelling cycle lasts 150–200 days and is primarily mediated by osteoblastic signals which promote the differentiation and maturation of osteoclast precursors. Activated osteoclasts create resorption pits with low pH to dissolve the inorganic matrix and lysomal enzymes, such as TRAP and cathepsin K, effectively digest the exposed type-1 collagen releasing specific degradation products. Osteoblasts are attracted to this eroded surface and begin to form new osteoid. Type-1 collagen, abundant in osteoblasts, is secreted as a procollagen precursor molecule into the extracellular space where it is cleaved at the amino- and carboxy-terminals releasing pro-peptides into the blood. Initially hydroxyapatite crystals are deposited in the osteoid then a slower mineralisation process continues over several months, followed by a period of quiescence. RANKL, an essential osteoclastogenic cytokine, is expressed on the surface of osteoblasts, it binds to its cellular receptor RANK on pre-osteoclasts and promotes their differentiation and activation. OPG a decoy receptor for RANKL, is secreted by osteoblasts and other stromal derived cells and reduces bone resorption by binding to RANK and preventing osteoclastic activity.

**Figure 2:**
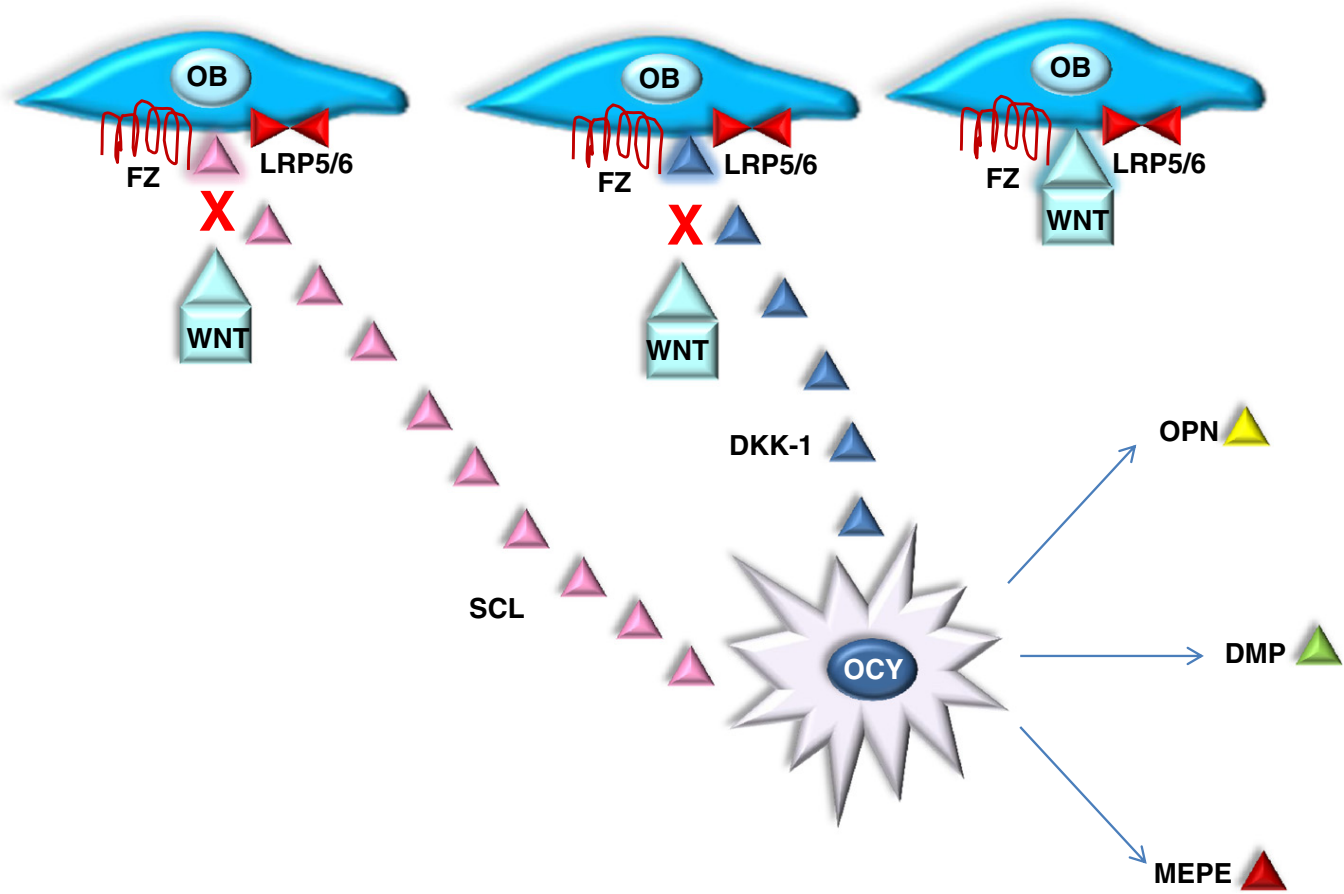
**Mechanism of blockade of the Wnt signalling pathway by osteocytes.** Osteocytes detect changes in bone morphology through their sensitivity to mechanical forces, thereby regulating bone turnover through direct physical contact with osteoblasts. Osteocytes produce OPN, DMP, MEPE, SCL and DKK-1. The β-catenin-dependent canonical Wnt signalling pathway controls gene expression by stabilizing β-catenin in regulating a diverse array of biological processes. It is initiated by binding of appropriate Wnt ligands to the frizzled (Fz) and low-density lipoprotein receptor-related proteins 5 and 6 (LRP-5/6) and can be antagonized by secreted proteins from SCL and the DKK family, that bind with high affinity to LRP-5 or LRP-6, thereby directly prevent Wnt binding. Wnt proteins act on osteoblast precursor cells through this pathway and promote their differentiation into mature osteoblasts. In addition, they can suppress bone resorption by up-regulating OPG and down-regulating RANKL expression in mature osteoblasts, leading to a net increase in bone mass [6]. Additionally research has targeted the complex regulation of osteocyte action by expression of PTH/PTHrP receptor’s (PPR’s). Osteocyte activation of PPR leads to down-regulation of Sost and increased Wnt signalling stimulating bone formation, accompanied by up-regulation of RANKL expression and osteoclast number increasing resorption. In contrast the main effect of PPR deletion on osteocytes is reduced osteoclast and osteoblast numbers and decreased bone remodelling [7].

Bone is a specialised connective tissue consisting primarily of glycoproteins and proteoglycans. The fibres of bone are mostly composed of type-I collagen impregnated with mineral in the form of hydroxyapatite. The functional integrity and strength of the skeleton is maintained by this highly cross-linked structure. Several factors may be involved in determining bone quality, including bone density and qualitative determinants of bone strength such as the rate of bone turnover, the extent of trabecular connectivity, cortical and periosteal bone size and skeletal morphometry [1]. Bone is metabolically active and is constantly being repaired and remodelled throughout an individual’s lifetime. Approximately twenty percent of bone tissue is replaced annually varying by site and type [2]. Remodelling begins before birth and continues until death, it is a highly synchronised process contained within basic multicellular units (Figure 1). Recent research has demonstrated the role of receptor activator of nuclear factor kappa B ligand/ receptor activator of nuclear factor kappa B/ osteoprotegerin (RANKL/RANK/OPG) in regulating bone metabolism [3]. Parathyroid hormone (PTH), PTH-related peptide (PTH-rP), 1,25-dihydroxyvitamin D_3_, prostaglandin E_2_, and interleukins among others regulate bone turnover through this system [4]. Additionally, bone metabolism is now known to be at least partly regulated by osteocytes, the fully differentiated osteoblasts present in lacunae in the mineralised matrix and osteoid tissue of bone [1]. Osteocytes detect mechanical loads and release signalling molecules (Figure 2) which coordinate the recruitment and activity of osteoblasts and osteoclasts thereby controlling bone turnover [5].

Under normal conditions bone formation and resorption are tightly linked through a variety of regulatory signals. Osteoporosis occurs when bone resorption is the more active resulting in a low bone mass and micro-architectural deterioration of bone tissue, leading to increased bone fragility and consequent increase in fracture risk. Osteoporotic fractures are a significant cause of morbidity and mortality, in the year 2010 there were an estimated 300,000 osteoporotic fractures in the UK and direct medical costs from such fractures were estimated at over two billion pounds [8]. Osteoporosis may be either primary (idiopathic) or secondary to a large number of conditions. These include hypogonadism, hyperthyroidism, skeletal metastases, multiple myeloma, anticonvulsant or oral corticosteroid use and alcohol abuse. Up to 30% of women and 55% of men with symptomatic vertebral crush fractures have an underlying cause of secondary osteoporosis [9]. The prevalence of osteoporosis increases with age, bone loss is reportedly more rapid in females in the first few years post menopause and is influenced by oestrogen deficiency [10], but it is also thought to increase in ageing men [11]. The World Health Organisation (WHO) has defined osteoporosis as a bone mineral density (BMD) measured by dual-energy X-ray absorptiometry (DXA) 2.5 standard deviations (SD) or more below the mean peak bone mass of premenopausal females (T-score ≤ −2.5 SD) [12]. Technical developments in the measurement of BMD have led to its adoption as the standard for diagnosis of osteoporosis, however the relatively poor sensitivity contrasting with high specificity means that many potential fractures will be missed if BMD assessment is used alone [13].

In recent years cellular components of the bone matrix have been identified and categorised as either markers of bone formation or resorption. Reliable, rapid, non-invasive, cost effective assays have been developed with improved sensitivity and specificity. Although these markers have been used in research for a long time they are only now being recognised as tools in the clinical management of bone disease. Technological advances have greatly enhanced the accuracy and reliability of bone marker measurement, although assays still vary significantly. In this review we will summarise the most widely used bone turnover makers (BTMs), briefly look at more novel markers and discuss their strengths, weaknesses and their clinical utility in the management of osteoporosis.

### Commonly used markers of bone turnover

Biomarkers of bone turnover can be measured in blood or urine and are used in selective combinations of formation and resorption markers that express the metabolic activity of osteoblasts or osteoclasts respectively, although in most circumstances the bone remodelling processes are coupled and tend to change in parallel. BTMs do not control skeletal metabolism and are not disease specific; they reflect the entire skeleton regardless of the underlying cause. Some markers represent both processes, e.g. osteocalcin (OC). Several of the available markers are non-specific, i.e. they are present in tissues other than bone and may therefore be influenced by non-skeletal processes [14]. Results should therefore always take into consideration the whole clinical picture and an understanding of the nature and source of each marker is essential for a comprehensive interpretation. The major advantages and disadvantages of each marker are included in Table 1.

**Table 1:** Major sources of variability in biochemical markers of bone turnover

**Bone marker (Abbreviation)**	**Source**	**Action**	**Advantages**	**Disadvantages**	**Analysis and sample type**
**Formation markers**					
Bone Alkaline Phosphatase (BAP)	Enzyme present in osteoblast plasma membranes	Enzymatic degradation of the mineralisation inhibitor pyrophosphate at alkaline pH	Low intra-individual variability <10% [15] Not affected by renal function [15] Food has little effect [16] Long circulatory half-life 1–2 days [17] Sample stability [18] Cheap	Up to 20% cross reactivity with liver isoforms [14] Changes with therapy minimal i.e. less than LSC of 25% [15] 2 peaks at 14:00 and 23:30 hrs Nadir 30% ↓at 06:30 [19] Multiple methodologies, can measure mass or activity [20]	Automated and manual immunoassays Serum, EDTA plasma
Osteocalcin (OC)	Major non-collagen bone Gla protein. Produced by osteoblasts during bone formation and bound to hydroxyapatite	Influences osteoid mineralisation Provides negative feedback during remodelling process	EDTA sample more stable [21] Late marker of osteoblast activity [15]	Intact molecule unstable [15] Large inter-lab variation [20] Released during formation and resorption [17] Short half-life of a few minutes [22,23] Influenced by Vit K status, renal function and circadian variability [15,17] OC gene regulated at transcriptional level by 1,25-OH_2_ Vit D Vit K essential co-factor for γ-carboxylation of OC resulting in ↑ affinity for Ca and hydroxyapatite [14]	Automated and manual immunoassays Multiplex microarray Serum, EDTA plasma
Procollagen type 1 Carboxy-terminal Propeptide (P1CP)	Specific product of proliferating osteoblasts and fibroblasts.	Cleaved from type 1 pro-collagen by proteases during type 1 collagen formation	Quantitative measure of newly formed type 1 collagen Thermostability [14]	Short half-life 6-8mins [14] Cleared in liver by mannose receptor so sensitive to thyroid hormones and IGF-1 [20] Highest levels 01:30 – 04:30, up to 20% higher than nadir 11:00 – 15:00 [19] Lacks sensitivity to changes during menopause [14]	Automated and manual immunoassays Serum, EDTA plasma
*Procollagen type 1 amiNo-terminal Propeptide (P1NP)	**Specific product of proliferating osteoblasts and fibroblasts.**	**Cleaved from type 1 pro-collagen by proteases during type 1 collagen formation**	**Low intra-individual variability **[20]** Small circadian rhythm **[15]** Stable at room temp **[21]** Good assay precision **[20]** Superior for PMO monitoring - change from baseline ↑up to 80% with anti-resorptive and ↓up to 200% with PTH medication within 3months **[15]	**Total assay affected by delayed clearance of monomeric fraction e.g. in renal failure or metastatic bone disease **[24]** Expensive**	**Automated and manual immunoassays Multiplex microarray Total or Intact fractions Serum, EDTA plasma**
**Resorption markers *Collagen derived***					
*Carboxy-Terminal cross-linked telopeptides of type 1 collagen (CTX)	**Type 1 collagen mainly bone Isomerisation to β aspartyl occurs in mature collagen**	**Cleaved from type 1 collagen by cathepsin-K during bone resorption**	**Variability↓ fasting **[25]** Sample stability, especially EDTA **[18][21]** Substantial ↓ post anti-resorptive treatment **[26]** Blood sample now preferential**	**Large circadian variation – highest 01:30 – 04:30 approx 2x nadir 11:00–15:00 **[27]	**Automated and manual immunoassays Multiplex microarray Urine, serum, EDTA plasma**
Carboxy-Terminal cross-linked telopeptides of type 1 collagen (ICTP or CTX-MMP)	Newly synthesised type 1 collagen predominantly bone	Cleaved from type 1 collagen by MMP during bone resorption		Large circadian variation [20] Influenced by renal and liver function [20] Not responsive to usual osteoporotic treatments [20]	Manual immunoassay
amiNo-Terminal cross-linked telopeptides of type 1 collagen (NTX)	Type 1 collagen mainly bone	Cleaved from type 1 collagen by cathepsin-K during bone resorption	Urine sample stable [15] uNTX sig predictor of fracture risk in postmenopausal women [28] Small dietary influence, although fasting blood sample preferred [15]	Large circadian variation Influenced by renal and liver function [20] Units based on manufacturer’s calibrator i.e. bone collagen equivalents [15]	Automated and manual immunoassays Urine, serum, EDTA plasma
Type 1 collagen alpha 1 helicoidal peptide (HELP)	Type 1 collagen Amino acid 620–633 sequence of the α chain	Cleaved from helical region of type 1 collagen by cathepsin-K during bone resorption	High correlation to other markers of collagen degradation [14]	24 hr collection – hard to collect 2^nd^ morning void with creatinine correction – additional analytical variability Clinical validity needs further investigation	Manual immunoassay Urinary marker
Deoxypyridinoline (DPD)	Mature type 1 collagen	Cross link released when mature type 1 collagen breaks down Mechanically stabilise the molecule	Reflect degradation of mature collagen only Specific to bone [14] Independent of dietary sources [20] Less invasive than blood	24 hr collection – hard to collect 2^nd^ morning void with creatinine correction – additional analytical variability Circadian variation [20]	Automated and manual immunoassays Urinary marker
Pyridinoline (PYD)	Mature type 1 and 11 collagen	Cross link released when mature collagen type 1 and 11 breaks down Mechanically stabilise the molecule	Reflect degradation of mature collagen only Independent of dietary sources [20]	Non-specific 24 hr collection – hard to collect 2^nd^ morning void with creatinine correction – additional analytical variability Circadian variation [20] Influenced by liver function [20]	Automated and manual immunoassays Urinary marker
**Resorption markers *Osteoclastic Enzymes***					
Tartrate Resistant Acid Phosphatase –isoform 5b (TRAP5b)	Isoform of acid phosphatase, resistant to tartrate, cleaved by proteases into 5b, present in ruffled border of osteoclasts	Cleaves type 1 collagen into fragments	Characteristic of osteoclastic activity [14]	Unstable at room temperature [22,23] Circadian variability ↑ immediately after exercise [29]	Automated and manual immunoassays Serum
Cathepsin K	Cysteine protease present in ruffled border of actively resorbing osteoclasts	Cleaves telopeptide and helical regions of type 1 collagen	Specific biomarker of osteoclastic activity [14]	Unstable at room temp Clinical validity needs further investigation	Manual immunoassay Serum, EDTA plasma
**Osteocyte activity markers**					
Receptor Activator of Nuclear factor Kappa B Ligand (RANKL)	Produced by osteoblasts, activated by B and T cells	Binds to RANK, which is expressed on osteoclasts and their precursors, stimulating their differentiation and activity	Novel biomarker Provide safety, efficacy and pharmacokinetics data to confirm drug mechanisms and mode of action for future use	Analytical problems Can measure free or OPG-bound [30] Circulating levels may not reflect bone microenvironment [31] Affected by thyroid function [32] Research method only Clinical and analytical validity needs further investigation	Manual research –grade immunoassay Total or soluble forms in serum
Osteoprotegerin (OPG)	Secreted by osteoblasts	Decoy receptor to RANKL reduces bone resorption by binding to RANK and preventing osteoclastogenesis	Novel biomarker Provide safety, efficacy and pharmacokinetics data to confirm drug mechanisms and mode of action for future use	Affected by thyroid function [32] Research method only Clinical and analytical validity needs further investigation	Manual research-grade immunoassay Serum
Dickkopf-related protein 1 (DKK1)	Produced by osteocytes	Inhibition of Wnt signalling pathway through binding to LRP5/6, blocking the Wnt effects on osteoblasts and decreasing bone formation	Key role in regulation of bone turnover	Research method only Clinical and analytical validity needs further investigation	Manual research –grade immunoassay Serum
Sclerostin (SCL)	Secreted by osteocytes	Inhibition of Wnt signalling pathway through binding to LRP5/6, blocking the Wnt effects on osteoblasts and decreasing bone formation	Significant ↓ with PTH therapy [33]	Research method only Affected by immobility [34] ↑ in type 1 and 2 diabetes [35,36] Clinical and analytical validity needs further investigation	Manual research-grade immunoassaySerum

*P1NP and CTX (highlighted in bold) are the markers of choice, recommended by the IOF, IFCC (2011) and NBHA (2012).

*ALP* alkaline phosphatase, *BSAP* bone specific alkaline phosphatase, *CTX* carboxy-terminal cross-linked telopeptides of type 1 collagen, *DKK-1* dickkopf-related protein 1, *DPD* deoxypyridinoline, *EDTA* ethylenediaminetetraacetic acid, *HELP* Type 1 collagen alpha 1 helicoidal peptide, *ICTP* carboxy-terminal cross-linked telopeptide of type 1 collagen, *IGF-1* insulin-like growth factor-1, *LRP* low-density lipoprotein receptor-related protein, *MMP* matrix metalloproteinases, *NTX* amino-terminal cross-linked telopeptide of type 1 collagen, *OPG* osteoprotegerin, *OC* osteocalcin, *PICP* procollagen type 1 carboxy-terminal propeptide, *PINP* procollagen type 1 amino-terminal propeptide, *PYD* pyridinoline, *RANK* receptor activator of nuclear factor kappa B, *RANKL* receptor activator of nuclear factor kappa B ligand, *SCL* sclerostin, *TRAP* tartrate resistant acid phosphatase.

#### Markers of bone formation

Markers of bone formation are either by-products of active osteoblasts expressed during the various phases of their development or osteoblastic enzymes. The most widely used markers of bone formation are measured in serum or plasma and include: bone specific alkaline phosphatase (BSAP), osteocalcin and the carboxy- and amino-terminal propeptides of type 1 collagen (P1CP, P1NP). P1NP has several functional advantages and has been recommended by the Bone Marker Standards Working Group; it has low inter-individual variability [20] and is relatively stable in serum at room temperature [21]. P1NP is cleared by liver endothelial cells via a macrophage receptor species, the scavenger receptor, that recognises and endocytoses modified proteins [37]. P1NP is released as a trimeric structure, but is rapidly broken down to a monomeric form by thermal degradation [38]. Current immunoassays detect either the trimeric ‘intact’ molecule (automated IDS iSYS assay) or can measure both fractions and are thus called ‘total’ P1NP assays (automated Roche Elecsys assay).

#### Markers of bone resorption

The majority of bone resorption markers are degradation products of bone collagen, the exception being tartrate-resistant acid phosphatase (TRAP5b). Earlier research into bone metabolism relied primarily on urinary markers such as pyridinoline (PYD) and deoxypyridinoline (DPD), which were time-consuming and cumbersome and relied on complete twenty-four hour urine collections or second morning void/ creatinine ratios, increasing the imprecision of the measurement. However, now that serum/ plasma markers are available these have become the preferred means of measuring resorption. Examples include carboxy-terminal and amino-terminal cross-linked telopeptide of type 1 collagen (CTX and NTX respectively), of which CTX is considered the marker of choice [20]. CTX is generated by cathepsin K activity, the CTX epitope contains an aspartyl-glycine motif that is susceptible to spontaneous isomerisation and racemisation generating four isoforms [17]; the α-aspartic acid converts to the β-form as the bone ages. Two automated immunoassays are available that target βCTX indicative of the breakdown of mature type 1 collagen (IDS iSYS and Roche Elecsys). The major disadvantage of CTX is its large circadian variation necessitating a morning fasting sample for accurate interpretation [25]. The choice of marker in clinical practice needs to be made on pragmatic grounds. Urine NTX may be the preferred marker in the clinic setting as unlike plasma CTX, it is not as sensitive to circadian changes and is not affected by food intake, it also avoids the invasive venepuncture associated with a blood sample and may be preferred by patients [39]. However the various drugs licensed for the treatment of osteoporosis have a differing spectrum of effects on BTMs and not all markers respond by the same amount for a given degree of bone resorption. Amongst the bone resorption markers, plasma CTX tends to change more than urine NTX which tends to change more than TRAP5b [20].

#### Markers of osteoclastogenesis

Osteoclast regulatory proteins are commonly measured in research, but have yet to find a niche clinically. The discovery of the OPG/RANK/RANKL system has clarified a major component of the bone remodelling cycle. RANKL is expressed *in vivo* in either membrane-bound or soluble form (sRANKL) and is also present in serum as a free or OPG-bound molecule, as a consequence design differences between immunoassays have created difficulties in comparing research and interpreting clinical data [30]. Furthermore circulating levels may not reflect the bone microenvironment [31]. Research into the relationship between circulating levels of OPG and sRANKL to BMD in postmenopausal osteoporosis are controversial, some studies reporting an inverse relationship [40], while others have found no association [41]. Rigorous testing of commercial assays and identification of the sources of variability are required before they can be adapted to routine clinical practice.

#### Osteocyte markers

Over the last decade research has focused mainly on the role of osteoclasts and osteoblasts in osteoporosis, more recently however, osteocytes have been found to play a key role in the regulation of bone turnover. Osteocytes are fully differentiated osteoblasts and lie in lacunae in the mineralized matrix and osteoid tissue of bone [42]. Osteocytes are able to detect changes in bone morphology, particularly micro-fractures through their sensitivity to mechanical forces, acting like bone mechanoreceptors [43]. They regulate bone turnover both through direct physical contact with other bone cells and by producing various factors which affect bone formation and can be measured in blood such as, sclerostin (SCL), dickkopf-related protein 1 (DKK1), dentin matrix protein 1 (DMP1) and matrix extracellular phosphoglycoprotein (MEPE).

DKK1 and SCL are secreted osteocyte markers acting as inhibitors to the Wnt signalling pathway through binding to low density lipoprotein receptor-related protein 5 and 6 (LRP5/6) and hence blocking the Wnt effects on osteoblasts decreasing bone formation (Figure 2) [44,45]. *In vivo* studies have shown that osteocyte depletion results in profound loss of trabecular bone mass [46-48] and suggest a close interaction between osteocytes and other bone cells, highlighting their role in the regulation of both bone formation and resorption.

Although widely used in research, their diagnostic importance remains to be validated due in part to their analytical and biological variability. In healthy adults, SCL levels correlate positively with age, BMI, and bone mineral content and negatively with osteocalcin and calcium [49]. SCL is increased in type 2 diabetes. Moreover, the transcriptional suppression of SCL production by PTH might be impaired in type 1 and type 2 diabetes [35]. SCL levels are significantly lower in osteoporotic compared to non-osteoporotic patients with type 2 diabetes [36]. The Wnt signalling pathway has recently been identified as central to the development of disuse osteoporosis [50]. Mechanical unloading in long-term immobilized patients causes up regulation of SCL and therefore inhibits bone formation via suppressed osteoblast activity and survival [34]. Circulating SCL reflects the severity of bone loss and is a candidate biomarker of osteoporosis severity in chronic spinal cord injury [51]. Higher serum SCL levels are associated with a greater risk of hip fractures in older women. In addition, the risk of hip fracture is amplified when high SCL levels are combined with lower BMD [52]. Serum SCL levels are regulated by both estrogens and PTH in postmenopausal women [53]. Serum SCL is decreased in women with postmenopausal osteoporosis compared with non-osteoporotic early postmenopausal women and positively correlated to lumbar spine BMD. Furthermore, levels are increased after 6 months treatment with risedronate, but remain essentially unchanged after 6 months teriparatide treatment [54]. However, serum or plasma SCL concentrations should be interpreted with caution as current assays produce very different results. Standardization of sclerostin assays is necessary before being introduced into general clinical laboratory use [55].

### Variability in markers of bone turnover

An understanding of the source and magnitude of the absolute inter and intra-person variability, including biological, pre-analytic and analytical variation, of each marker is necessary to interpret serial measurements and individualise treatment.

#### Biological variability

##### Intra-individual variation

Bone turnover shows a circadian rhythm, this is more obvious in the serum and urinary markers of bone resorption. βCTX for example is highest between 01:30 and 04:30 hours and may be more than twice that at the nadir between 11:00 and 15:00 hours [27], this may be attenuated by several factors such as; age, gender, ethnicity, menopausal status, osteoporotic stage and anti-resorptive agents or calcium supplementation [19], but the disparity is diminished with fasting [25]. All bone markers are significantly lower in the fed state with the exception of BSAP, this may be due to several factors including the clearance rate of individual markers or food composition [16] and may be partly explained by variation in serum insulin [25]. Osteocalcin and P1CP follow the same diurnal pattern but show only twenty percent difference and BSAP has two peaks at 14:00 and 23:30 hours with a nadir thirty percent reduced at 06:30 [19]. Therefore timing of the sample collection and fasting status should be tightly controlled.

The existence of intra-individual low-frequency biological rhythms, imply that biomarkers can also vary between consecutive days, this is more noticeable in the urinary resorption markers [14]. There is a degree of controversy regarding seasonal variation with some researchers suggesting that overall seasonal changes are insignificant [56], whilst others have found a substantial wintertime increase [57], which may be due in part to reduced levels of vitamin D. Physical activity is also significant, TRAP and to a lesser extent BSAP and CTX are reduced immediately after plyometrics, but return to pre concentrations within two hours. Interestingly similar changes were found in PTH [29]. Details of exercise in the previous twenty-four hours should therefore be recorded.

Bone turnover varies with the menstrual cycle, research suggests that osteoblastic activity is higher during the luteal period [58] and bone resorption is increased during the follicular phase [59]. Pregnancy affects all BTMs due in part to the calcium requirements of the foetus, but also to changes in maternal glomerular filtration rate (GFR) affecting renal clearance. However the time change is contentious, one study following ten women at regular intervals reported an increase in urinary resorption markers throughout pregnancy with a significant increase in bone formation in the third trimester [60]. A more recent study measured serum OPG, RANKL, osteocalcin and CTX in twenty six different women at each trimester. The study found increased bone formation in the first trimester and increased resorption in the second which surprisingly decreased again in the third trimester [61]. Postpartum, levels gradually start to decrease but may still be higher than pre-pregnancy levels for up to a year [62].

A comprehensive drug history should also be taken into account when interpreting bone marker results. Anti-resorptive drugs such as bisphosphonates [26] and hormone replacement therapy (HRT) [63] have a major effect on markers of bone resorption and long-term corticosteroid therapy is known to suppress bone formation [64].

Inflammatory conditions are major precipitators for bone loss, especially rheumatoid arthritis (RA) which is further aggravated by decreased functional activity and the use of glucocorticoids [65]. In a prior study, we found that B-cell depletion increases bone formation and decreases bone resorption in RA patients. This may be a direct effect on osteoblasts and osteoclasts respectively and be at least partially explained by the decreased inflammation and disease activity [66]. In diabetes serum osteocalcin is negatively correlated with glucose levels and advanced glycation end products (AGEs) are known to have a negative impact on bone [67]. Thyroid disorders such as thyrotoxicosis are well known to affect bone turnover. Thyroid stimulating hormone (TSH) receptors are present in both osteoblasts and osteoclasts and the low TSH levels observed in thyroidectomised patients on L-thyroxine are associated with an increase in OPG and decrease in RANKL and are significantly correlated with vertebral fractures [32]. Bone markers are cleared through the liver or kidneys and are also influenced by diseases affecting these systems, decreased GFR for example will decrease the urinary excretion of CTX and therefore increase serum levels. They are also affected by any disease states leading to increased periods of bed rest and immobility. Research has shown that microgravity induces significant and progressive bone loss, a consequence of increased bone resorption and retardation of bone formation [68]. Certainly levels of all bone markers increase significantly in the first few weeks after fracture and may remain elevated for up to a year. The rate of increase is dependent on the location, severity and size of the fracture and the age of the patient. BTM’s can be elevated for up to 6 months after minor fractures e.g. forearm fractures but up to one year after a hip fracture and needs to be taken into consideration when measuring them [19,69]. However, they fall gradually over time and using a reduction of 50% in bone resorption when using anti-resorptives as a good indicator of response would be greater than any reduction that might otherwise occur.

In light of the above evidence it can be seen that to use bone turnover to monitor change can be quite difficult. In order to minimise problems it is best to measure the BTMs in as similar a set of circumstances as possible. Particular attention should be paid to the time of day and hence research studies tend to use early morning fasted samples. One way in which to help overcome within person variability in serial measurements and to monitor therapy is to use the ‘least significant change’ (LSC) model [70]. LSC at a significance level of p=<0.05 is defined as 1.96*√2*√ (CV_1_^2^+CV_A_^2^); where CV_1_ is the within-subject coefficient of variation and CV_A_ is the total analytical imprecision. LSC identifies the true physiological change in the marker. In general a change of more than twenty percent is considered significant for formation markers [71], similarly between twenty-seven to thirty-six percent is significant for markers of bone resorption [72].

##### Inter-individual variation

**Figure 3:**
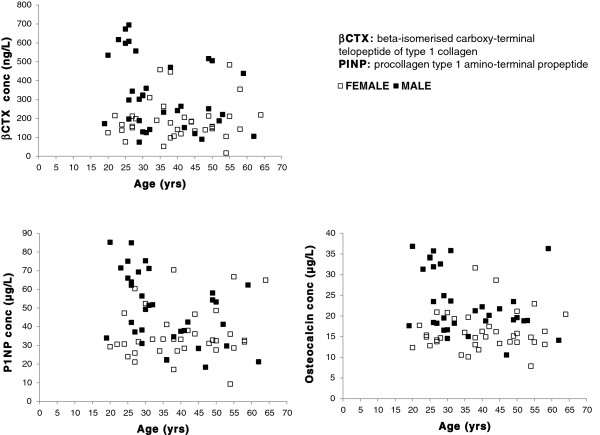
**Age related changes to bone turnover markers.** Blood samples from seventy healthy volunteers (33 males aged 19 to 62 years and 37 females aged 20 to 64 years), collected between 10–11 am, were analysed on the Elecsys 2010 (Roche, Lewes UK), for βCTX, P1NP and osteocalcin to verify the effect of age on bone turnover.

Between person variability is much harder to control, e.g. age, gender, and menopausal status, but is equally important to validate results. Bone metabolism rates are higher in infants up to three years of age, they are relatively stable throughout adolescence but sex-specific increases in bone marker levels are evident during the pubertal growth spurt and are reportedly influenced by pubertal stage rather than age [73]. BTMs are higher in men between twenty and thirty years of age then reach their lowest levels during their fifties [14], whereas in females there is a substantial increase in bone turnover corresponding to oestrogen deficiency during the menopause [28]. We checked our local population and found a trend towards higher bone turnover in males during their third decade whilst reaching peak bone mass, although there seemed to be little evidence of any age related change in women possibly due to the lower numbers of postmenopausal females in our cohort (Figure 3). It is important for each laboratory to investigate the transferability of the quoted reference intervals to its own patient population based on equivalent standardised collection conditions. The widespread availability of automated immunoassays now means that harmonisation of method specific reference ranges is possible and studies from well-characterised populations have reported robust BTM ranges in large well defined cohorts [74]. In contrast to the use of reference ranges some researchers have suggested combining a marker of formation and resorption to gain a direct insight into the changes in the balance and rate of bone turnover in relation to a reference value [75], leading factors in estimating fracture risk and prognosis. Given the large observed differences observed between genders, different ages and developmental stages means that care must be taken when comparing populations and in the design of research studies.

#### Analytical variability

##### Technical variation

Over the last decade many of the traditional BTM immunoassays have been automated, improving technical performance and increasing their availability. Nevertheless, analytical aspects such as within and between batch precision, accuracy and standardisation, remain problematic. Inter-laboratory variation is also crucial; a European study in 2001, measuring pooled samples of serum and urine in seventy-three laboratories concluded that even with identical assays results for the majority of the markers were significantly different [76]. Similarly an American study in 2010 comparing six commercial laboratories over an eight month period concluded that reproducibility varied substantially for urine NTX and serum BSAP [77]. Moreover there is an extensive list of bone markers being offered making it very difficult to compare research evidence. Consequently, the International Osteoporosis Foundation (IOF), the International Federation of Clinical Chemistry and Laboratory Medicine (IFCC) [20] and the National Bone Health Alliance (NBHA) [78], have recommended that a marker of bone formation and resorption, namely P1NP and CTX, are used as reference analytes in clinical studies. They go on to stipulate that these markers should be measured by standardised assays to minimise immunochemical heterogeneity and recommend that manufacturers adopt international reference standards and minimise batch to batch variability [20].

##### Sample stability

Appropriate control of sample collection and preparation is vital for successful BTM measurement. Several BTMs, especially osteocalcin and TRAP5b, are sensitive to thermo degradation and levels can be significantly reduced after only a few hours at room temperature [22,23]. TRAP5b activity is also reduced during storage, samples must be kept at −70°C or lower and multiple freeze-thaw cycles should be avoided. No significant decrease has been detected in CTX stored at −20°C or lower for up to three years, nevertheless it rapidly decreases in serum at both 4°C and 37°C. The molecular mechanism is unknown but this decrease is minimised by ethylenediaminetetraacetic acid (EDTA) [18]. CTX is reportedly stable in EDTA blood tubes before separation for up to forty-eight hours, likewise osteocalcin becomes stable for up to eight hours at room temperature [21]. Consequently blood should be collected into EDTA tubes and separated as soon as possible, if samples are not analysed immediately they should be stored at −20°C or lower. Both P1NP and BSAP were found to be stable in any sample type [21]. Notably current TRAP5b assays are not affected by haemolysis, but erythrocytes are known to contain proteases which degrade osteocalcin. Grossly haemolysed samples in general should always be avoided.

### Clinical usefulness of bone turnover markers in osteoporosis

BTMs are frequently used in clinical trials and provide valuable information on the efficacy of osteoporotic treatments, but their predictive value is limited by their large biological variation and diagnostically they are less often used for individualized patient care. Other routine laboratory investigations are frequently used to identify or exclude secondary causes of osteoporosis such as hyperparathyroidism, vitamin D status, thyrotoxicosis and hypogonadism [79].

#### Diagnostic

Currently the WHO recommends the use of BMD of the spine and proximal femur measured by DXA as the gold standard to diagnose osteoporosis and its severity [12]. Although BMD has methodological limitations especially in the elderly due in part to degenerative changes in the lumbar spine [80], BTMs alone would not be suitable to estimate bone loss.

#### Prediction of bone loss

Women generally lose about one to two percent of their bone per year after the menopause, however thirty percent lose bone at a faster rate [28]. Longitudinal studies of post-menopausal women have demonstrated two characteristic groups; high bone turnover and normal or low bone turnover. Serial BTM measurements are effective in identifying those women who lose bone most rapidly, this is important because this group respond more readily to anti-resorptive medication [81]. Furthermore a meta-analysis of eighty-five studies reported a significant correlation between serial levels of BTMs and BMD measurements during bisphosphonate treatment [82], the association becomes stronger with advancing age [83]. However, BTMs should only be used to supplement BMD not measured in isolation.

#### Prediction of fracture risk

BMD is widely used to predict osteoporotic fractures but approximately thirty to fifty percent of patients with fragility fractures have T-scores above the osteoporotic threshold [84]. There is evidence that high bone turnover, as measured by a single or combination of BTMs, is associated with an increased fracture risk [28], but their use alone to predict fracture has yet to be established. Two clinical risk assessment algorithms have been validated for use in the UK to predict fracture incidence over ten years [85], namely FRAX and QFracture, currently they do not include all risk factors. BTMs have not been included because of their inconsistency in research studies so far. There is a need for studies confirming whether the addition of BTMs to FRAX would result in increased sensitivity and specificity.

#### Treatment selection and monitoring

BMD and BTMs are independent predictors of fracture risk, recent evidence does not support the use of BTMs to select the optimal treatment, but BTMs can be used to monitor treatment efficacy before BMD changes can be evaluated. Additionally early changes in BTMs can be used to measure the clinical efficacy of an anti-resorptive treatment and to reinforce patient compliance [26]. The effectiveness of osteoporotic therapy can be assessed by serial BMD measurements usually by DXA, but quantifiable changes in bone mass are small and are only apparent after twelve to twenty-four months, furthermore they only measure net balance in a very small portion of the skeleton. DXA reproducibility is also affected by machine and operator error plus patient variability (weight or degenerative changes) [86]. The minimum acceptable precision for an individual technician is 1.9% (LSC 5.3%) at the lumbar spine and 1.8% (LSC 5.0%) at the total hip. Intervals between measurements depend on the patient’s clinical status, but given the need to exceed the LSC and the relatively modest changes in BMD observed with most treatments it is generally going to be a minimum of two years before a significant change can be observed. Indeed, there are trends for a variety of reasons towards less frequent measurement of BMD to three or even five year intervals [87].

Meta-regression analysis has found no evidence of a relationship between BMD changes and reduction in risk of fractures among patients receiving calcium with or without vitamin D supplementation. Calcium and/or Vitamin D may reduce fracture rates through a mechanism independent of bone density [13]. BTMs on the other hand show a substantial and more immediate global effect, they measure both bone formation and resorption rate and can classify patients into low or high remodelling groups. Osteoporosis treatments such as bisphosphonates, strontium ranelate, denosumab, hormone replacement therapy (HRT) and selective estrogen receptor moderators (SERMs) act by reducing BTM levels by forty to sixty percent within three to six months [88]. Thus one use of BTMs is to give an early indication of the success of the treatment. Baseline measurements can be repeated at the next follow up appointment say three to six months later. A significant change in BTMs as assessed by the LSC method can then be used to judge success of the treatment and will hopefully be reflected by an increase in BMD in the fullness of time. In the meantime the change in BTM supplies reassurance to the clinician and can be used to encourage the patient. Unfortunately, as BTMs are highly variable this is at best only an indication.

There has been considerable discussion about how long to treat with bisphosphonates, because these drugs accumulate in the skeleton, leading to a reservoir that continues to be released for months or years after treatment has stopped. These medications also result in a low bone turnover state over time with both resorption and formation reduced. This combined with concerns over microfracture, the possibility that they may prevent bone healing and the association with atypical femoral shaft fractures has led to the belief that it may not be wise to continue these medications indefinitely. It is generally accepted that the need to continue bisphosphonates be reviewed after 5 years and kept under review until ten years of treatment. Depending on the individual circumstances a decision to stop treatment, give a drug holiday or change treatment may be made. If a drug holiday is decided upon then BTMs could be checked at regular intervals, e.g. annually. Once these are rising again and especially on return to pre-treatment levels therapy could be restarted. Such an approach may be particularly useful with longer acting agents such as zoledronic acid [89]. The BTMs should be used in conjunction with the clinical circumstances and with repeated BMD after appropriate time intervals.

More recently anabolic agents such as PTH, e.g. teriparatide, have become available which stimulate osteoblastic activity. Markers of bone formation increase early after the initiation of teriparatide therapy with a delayed, but significant, increase in resorption markers [90]. It has been proposed clinically to measure P1NP at baseline and three months post treatment a positive response is defined as a change of greater than 10 μg/L [91].

## Conclusions

During the last decade significant advances have been made in the identification and characterisation of specific BTMs for use in clinical drug trials and to aid in the therapeutic management of osteoporosis. Technological developments have greatly enhanced assay performance producing reliable, rapid, non-invasive cost effective assays with improved sensitivity and specificity. We now have a greater understanding of the need to regulate pre-analytical sample collection to minimise the effects of biological variation. The use of BTMs to select those at risk of fractures is not routinely recommended partly due to their degree of variability. However, baseline measurements of resorption markers are useful before commencement of anti-resorptive treatment e.g. bisphosphonates or denosumab and can be checked 3–6 months later to check response and adherence to treatment. Similarly a formation marker such as P1NP can be used to monitor bone forming agents such as PTH analogues. BTMs may also be useful when monitoring patients during treatment holidays and aid in the decision as to when therapy should be recommenced. The recent recommendations by the Bone Marker Standards Working Group aim to standardise research and include a marker of bone resorption (CTX) and formation (P1NP) in all future studies. They anticipate that manufacturers will calibrate their assays in future using an international reference standard to establish robust reference ranges. It is hoped that improved research in turn will lead to optimise markers for the clinical management of osteoporosis and other bone diseases. The biochemical assessment, utilizing BSAP, is now the mainstay of the diagnosis and management of metabolic bone disease in chronic kidney disease.

## Abbreviations

AGEs: Advanced glycation end products; ALP: Alkaline phosphatase; BSAP: Bone specific alkaline phosphatase; βCTX: Beta-isomerised carboxy terminal telopeptide of type I collagen; BMD: Bone mineral density; BTM: Bone turnover marker; CTX: Carboxy-terminal cross-linked telopeptides of type 1 collagen; CVA: Total analytical imprecision; CV1: Within-subject coefficient of variation; DKK-1: Dickkopf-related protein 1; DMP1: Dentin matrix protein 1; DPD: Deoxypyridinoline; DXA: Dual-energy X-ray absorptiometry; EDTA: Ethylenediaminetetraacetic acid; Fz: Frizzled protein; GFR: Glomerular filtration rate; GH: Growth hormone; HELP: Type 1 collagen alpha 1 helicoidal peptide; HRT: Hormone replacement therapy; ICTP: Carboxy-terminal cross-linked telopeptide of type 1 collagen; IDS: Immuno-Diagnostic Systems Ltd; IFCC: International Federation of Clinical Chemistry and Laboratory Medicine; IGF1: Insulin-like growth factor 1; IOF: International Osteoporosis Foundation; LRP: Low-density lipoprotein receptor-related protein; LSC: Least significant change; MEPE: Matrix extracellular phosphoglycoprotein; MMP: Matrix metalloproteinases; MoM: Multiple of the median; MoMF: Multiple of the median formation marker; MoMR: Multiple of the median resorption marker; NBHA: National Bone Health Alliance; NTX: Amino-terminal cross-linked telopeptide of type 1 collagen; OB: osteoblast; OC: osteocalcin; OCY: osteocyte; OPG: osteoprotegerin; OPN: osteopontin; PICP: Procollagen type 1 carboxy-terminal propeptide; PINP: Procollagen type 1 amino-terminal propeptide; PPR: PTH/PTHrP receptor; PTH: Parathyroid hormone; PTHrP: Parathyroid hormone related peptide; PYD: pyridinoline; RA: Rheumatoid arthritis; RANK: Receptor activator of nuclear factor kappa B; RANKL: Receptor activator of nuclear factor kappa B ligand; sRANKL: Soluble receptor activator of nuclear factor kappa B ligand; SCL: sclerostin; SD: Standard deviation; SERMs: Selective estrogen receptor moderators; Sost: SclerOSTeosis gene; TRAP: Tartrate resistant acid phosphatase; TSH: Thyroid stimulating hormone; UK: United Kingdom; WHO: World Health Organisation; Wnt: Wingless and Integration-1.

## Competing interests

JMvL has received a research grant, consultancy and speaker fees from Roche. SPT has received speaker fees from Ely Lilly.

## Author’ contributions

GW drafted the manuscript. ME revised the manuscript. SPT and HKD revised and critically appraised the manuscript. JMvL revised, critically appraised and provided overall supervision for the project. All authors read and approved the final manuscript.

## References

[B1] Datta HK Ng FW Walker JA Tuck SP Varanasi SS The cell biology of bone metabolism-a review J Clin Path 2008 61 577 587 10.1136/jcp.2007.048868 18441154

[B2] Carey JJ Licata AA Delaney MF Biochemical markers of bone turnover Clin Rev Bone Miner Metab 2006 4 3 197 212 10.1385/BMM:4:3:197

[B3] Vega D Maalouf NM Sakhaee K The role of receptor activator of nuclear factor-κB (RANK)/RANK Ligand/Osteoprotegerin: clinical implications J Clin Endocrinol Metab 2007 92 12 4514 4521 10.1210/jc.2007-0646 17895323

[B4] Leibbrandt A Penninger JM RANK/RANKL: regulators of immune responses and bone physiology Ann N Y Acad Sci 2008 1143 123 150 10.1196/annals.1443.016 19076348

[B5] Klein-Nulend J Bakker AD Bacabac RG Vatsa A Weinbaum S Mechanosensation and transduction in osteocytes Bone 2013 54 2 182 190 10.1016/j.bone.2012.10.013 23085083

[B6] Kobayashi Y Maeda K Takahashi N Roles of Wnt signalling in bone formation and resorption Jpn Dent Sci Rev 2008 44 76 82 10.1016/j.jdsr.2007.11.002

[B7] Bellido T Saini V Pajevic PD Effects of PTH on osteocyte function Bone 2013 54 2 250 257 10.1016/j.bone.2012.09.016 23017659 PMC3552098

[B8] National osteoporosis societyhttp://www.nos.org.uk/page.aspx?pid=328

[B9] Tuck SP Francis RM Osteoporosis Postgrad Med J 2002 78 526 532 10.1136/pmj.78.923.526 12357012 PMC1742482

[B10] Bongartz TA Scholmerich J Straub RH Maricic M, Gluck OS From Osteoporosis in postmenopausal women Bone disease in rheumatology 2005 Arizona: Lippincott Williams and Wilkens 155 156

[B11] Bauer D Garnero P Harison SL Cauley JA Eastell R Ensrud KE Orwoll E Biochemical markers on bone turnover, hip loss and fracture in older men: the MrOS study J Bone Miner Res 2009 24 12 2032 2038 10.1359/jbmr.090526 19453262 PMC2791517

[B12] Kanis JA McCloskey EV Johansson H Oden A Melton LJ Khaltaev N A reference standard for the description of osteoporosis Bone 2008 42 467 475 10.1016/j.bone.2007.11.001 18180210

[B13] Rabinda V Bruyère O Reginster JY Relationship between bone mineral density changes and risk of fractures among patients receiving calcium with or without vitamin D supplementation: a meta-regression Osteoporos Int 2011 22 893 901 10.1007/s00198-010-1469-x 21060990

[B14] Seibel MJ Biochemical markers of bone turnover: part 1: biochemistry and variability Clin Biochem Rev 2005 26 97 122 16648882 PMC1320175

[B15] Brown JP Albert C Nassar BA Adachi JD Cole D Davison KS Dooley KC Don-Wauchope A Douville P Hanley DA Jamal SA Josse R Kaiser S Krahn J Krause R Kremer R Lepage R Letendre E Morin S Ooi DS Papaioaonnou A Ste-Marie L-G Bone turnover markers in the management of osteoporosis Clin Biochem 2009 42 929 942 10.1016/j.clinbiochem.2009.04.001 19362543

[B16] Clowes JA Hannon RA Yap TS Hoyle NR Blumsohn A Eastell R Effect of feeding on bone turnover markers and its impact on biological variability of measurements Bone 2002 30 6 886 890 10.1016/S8756-3282(02)00728-7 12052458

[B17] Swaminathan R Biochemical markers of bone turnover Clin Chim Acta 2001 313 95 105 10.1016/S0009-8981(01)00656-8 11694245

[B18] Qvist P Munk M Hoyle N Christiansen C Serum and plasma fragments of C-telopeptides of type I collagen (CTX) are stable during storage at low temperatures for 3 years Clin Chim Acta 2004 350 1–2 167 173 15530474 10.1016/j.cccn.2004.07.024

[B19] Hannon R Eastell R Preanalytical variability of biochemical markers of bone turnover Osteoporos Int 2000 11 Suppl 6 S30 44 11193238 10.1007/s001980070004

[B20] Vasikaran S Eastell R Bruyère O Foldes AJ Garnero P Griesmacher A McClung M Morris HA Silverman S Trenti T Wahl DA Cooper C Kanis JA for the IOF-IFCC Bone Marker Standards Working Group Markers of bone turnover for the prediction of fracture risk and monitoring of osteoporosis treatment: a need for international reference standards Osteoporos Int 2011 22 391 420 10.1007/s00198-010-1501-1 21184054

[B21] Stokes FJ Ivanov P Bailey LM Fraser WD The effects of sampling procedures and storage conditions on short-term stability of blood-based biochemical markers of bone metabolism Clin Chem 2011 57 1 138 140 10.1373/clinchem.2010.157289 20974796

[B22] Blumsohn A Hannon RA Eastell R Apparent instability of osteocalcin in serum as measured with different commercially available immunoassays Clin Chem 1995 41 318 319 7874787

[B23] Halleen JM Alatalo SL Suominen H Cheng S Janckila AJ Väänänen HK Tartrate-resistant acid phosphatase 5b: a novel serum marker of bone resorption J Bone Miner Res 2000 15 7 1337 1345 10.1359/jbmr.2000.15.7.1337 10893682

[B24] Marin L Koivula M-K Jukkola-Vuorinen A Leino A Risteli J Comparison of total and intact aminoterminal propeptide of type 1 procollagen assays in patients with breast cancer with or without bone metastases Ann Clin Biochem 2011 48 447 451 10.1258/acb.2011.011040 21733929

[B25] Bjarnason NH Henriksen EEG Alexandersen P Christgau S Henriksen DB Christiansen C Mechanism of circadian variation in bone resorption Bone 2002 30 307 313 10.1016/S8756-3282(01)00662-7 11792602

[B26] Bergmann P Body JJ Boonen S Boutsen Y Devogelaer JP Goemaere S Kaufman JM Reginster JY Gangji V Members of the Advisory Board on Bone Markers Evidence-based guidelines for the use of biochemical markers of bone turnover in the selection and monitoring of bisphosphonate treatment in osteoporosis: a consensus document of the Belgian bone club Int J Clin Pract 2009 63 1 19 26 10.1111/j.1742-1241.2008.01911.x 19125989 PMC2705815

[B27] Wichers M Schmidt E Bidlingmaier F Klingmüller D Diurnal rhythm of cross laps in human serum Clin Chem 1999 45 1858 1860 10508135

[B28] Garnero P Sornay-Rendu E Claustrat B Delmas PD Biochemical markers of bone turnover, endogenous hormones and the risk of fractures in postmenopausal women: the OFELY study J Bone Miner Res 2000 15 1526 1536 10.1359/jbmr.2000.15.8.1526 10934651

[B29] Rogers RS Dawson AW Wang Z Thyfault JP Hinton PS Acute response of plasma markers of bone turnover to a single bout of resistance training or plyometrics J Appl Physiol 2011 111 1353 1360 10.1152/japplphysiol.00333.2011 21868687

[B30] Bowsher RR Sailstad JM Insights in the application of research-grade diagnostic kits for biomarker assessments in support of clinical drug development: Bioanalysis of circulating concentrations of soluble receptor activator of nuclear factor κB ligand J Pharm Biomed Anal 2008 48 5 1282 1289 10.1016/j.jpba.2008.09.026 18977625

[B31] Kearns AE Khosla S Kostenuik PJ Receptor activator of nuclear factor κB ligand and osteoprotegerin regulation of bone remodelling in health and disease Endocr Rev 2008 29 2 155 192 18057140 10.1210/er.2007-0014 PMC2528846

[B32] Nicholls JJ Brassill MJ Williams GR Duncan Bassett JH The skeletal consequences of thyrotoxicosis J Endocrinol 2012 213 3 209 221 10.1530/JOE-12-0059 22454529

[B33] Drake MT Srinivasan B Mödder UI Peterson JM McCready LK Riggs BL Dwyer D Stolina M Kostenuik P Khosla S Effects of parathyroid hormone treatment on circulating sclerostin levels in postmenopausal women J Clin Endocrinol Metab 2010 95 11 5056 5062 10.1210/jc.2010-0720 20631014 PMC2968729

[B34] Gaudio A Pennisi P Bratengeier C Torrisi V Lindner B Mangiafico RA Pulvirenti I Hawa G Tringali G Fiore CE Increased sclerostin serum levels associated with bone formation and resorption markers in patients with immobilization-induced bone loss J Clin Endocrinol Metab 2010 95 5 2248 2253 10.1210/jc.2010-0067 20305005

[B35] Gennari L Merlotti D Valenti R Ceccarelli E Ruvio M Pietrini MG Capodarca C Franci MB Campagna MS Calabrò A Cataldo D Stolakis K Dotta F Nuti R Circulating sclerostin levels and bone turnover in type 1 and type 2 diabetes J Clin Endocrinol Metab 2012 97 5 1737 1744 10.1210/jc.2011-2958 22399511

[B36] Garcia-Martin A Rozas-Moreno P Reyes-Garcia R Morales-Santana S Garcia-Fontana B Garcia-salcedo JA Muñoz-Torres M Circulating levels of sclerostin are increased in patients with type 2 diabetes mellitus J Clin Endocrinol Metab 2012 97 1 234 241 10.1210/jc.2011-2186 22031520

[B37] Melkko J Hellevik T Risteli L Risteli J Smedsrød S Clearance of NH_2_-terminal propeptides of types I and III Procollagen is a physiological function of the scavenger receptor in liver endothelial cells J Exp Med 1994 179 405 412 10.1084/jem.179.2.405 8294857 PMC2191385

[B38] Brandt J Krogh TN Jensen CH Frederiksen JK Teisner B Thermal instability of the trimeric structure of the N-terminal propeptide of human Procollagen type I in relation to assay technology Clin Chem 1999 45 1 47 53 9895337

[B39] Baxter I Rogers A Eastell R Peel N Evaluation of urinary N-telopeptide of type I collagen measurements in the management of osteoporosis in clinical practice Osteoporos Int 2013 24 941 947 10.1007/s00198-012-2097-4 22872068

[B40] Jabbar S Drury J Fordham JN Datta HK Francis RM Tuck SP Osteoprotegerin, RANKL and bone turnover in postmenopausal osteoporosis J Clin Pathol 2011 64 354 357 10.1136/jcp.2010.086595 21307155

[B41] Liu JM Zhao HY Ning G Zhao YJ Chen Y Zhang Z Sun LH Xu M-Y Chen JL Relationships between the changes of serum levels of OPG and RANKL with age, menopause, bone biochemical markers and bone mineral density in Chinese women aged 20–75 Calcif Tissue Int 2005 76 1 1 6 10.1007/s00223-004-0007-2 15455183

[B42] Noble BS The osteocyte lineage Arch Biochem Biophys 2008 473 2 106 11 10.1016/j.abb.2008.04.009 18424256

[B43] Bonewald LF Osteocytes: a proposed multifunctional bone cell J Musculoskelet Neuronal Interact 2002 2 3 239 41 15758443

[B44] Zhang Y Wang Y Li X Zhang J Mao J Li Z Zhang J Li L Harris S Wu D The LRP5 high-bone-mass G171V mutation disrupts LRP5 interaction with Mesd Mol Cell Biol 2004 24 11 4677 4684 10.1128/MCB.24.11.4677-4684.2004 15143163 PMC416395

[B45] Li X Zhang Y Kang H Liu W Liu P Zhang J Harris SE Wu D Sclerostin binds to LRP5/6 and antagonizes canonical Wnt signalling J Biol Chem 2005 280 19883 19887 10.1074/jbc.M413274200 15778503

[B46] You L Temiyasathit S Lee P Kim CH Tummala P Yao W Kingery W Malone AM Kwon RY Jacobs CR Osteocytes as mechanosensors in the inhibition of bone resorption due to mechanical loading Bone 2008 42 1 172 179 10.1016/j.bone.2007.09.047 17997378 PMC2583402

[B47] Gross TS King KA Rabaia NA Pathare P Srinivasan S Upregulation of osteopontin by osteocytes deprived of mechanical loading or oxygen J Bone Miner Res 2005 20 2 250 256 15647819 10.1359/JBMR.041004 PMC1435734

[B48] Noble BS Peet N Stevens HY Brabbs A Mosley JR Reilly GC Reave J Skerry TM Lanyon LE Mechanical loading: biphasic osteocyte survival and targeting of osteoclasts for bone destruction in rat cortical bone Am J Physiol Cell Physiol 2003 284 C934 C943 10.1152/ajpcell.00234.2002 12477665

[B49] Amrein K Amrein S Drexler C Dimai HP Dobnig H Pfeifer K Tomaschitz A Pieber TR Fahrleitner-Pammer A Sclerostin and its association with physical activity, age, gender, body composition and bone mineral content in healthy adults J Clin Endocrinol Metab 2012 97 1 148 154 10.1210/jc.2011-2152 21994959

[B50] Lin C Jiang X Dai Z Guo X Weng T Wang J Li Y Feng G Gao X He L Sclerostin mediates bone response to mechanical unloading through antagonizing Wnt/β-Catenin signalling J Bone Miner Res 2009 24 10 1651 1661 10.1359/jbmr.090411 19419300

[B51] Morse LR Sudhakar S Lazzari AA Tun C Garshick E Zafonte R Battaglino RA Sclerostin: a candidate biomarker of SCI-induced osteoporosis Osteoporos Int 2013 24 961 968 10.1007/s00198-012-2072-0 22801952 PMC3611240

[B52] Arasu A Cawthon PM Lui L-Y Do TP Arora PS Cauley JA Ensrud KE Cummings SR Serum sclerostin and risk of hip fracture in older Caucasian women J Clin Endocrinol Metab 2012 97 6 2027 2032 10.1210/jc.2011-3419 22466341 PMC3387417

[B53] Mirza FS Padhi ID Raisz LG Lorenzo JA Serum sclerostin levels negatively correlate with parathyroid hormone levels and free estrogen index in postmenopausal women J Clin Endocrinol Metab 2010 95 4 1991 1997 10.1210/jc.2009-2283 20156921 PMC2853994

[B54] Polyzos SA Anastasilakis AD Bratengeier C Woloszczuk W Papatheodorou A Terpos E Serum sclerostin levels positively correlate with lumbar spinal bone mineral density in postmenopausal women – the six-month effect of risedronate and teriparatide Osteoporos Int 2012 23 1171 1176 10.1007/s00198-010-1525-6 21305266

[B55] McNulty M Singh RJ Li X Bergstralh EJ Kumar R Determination of serum and plasma sclerostin concentrations by enzyme-linked immunoassays J Clin Endocrinol Metab 2011 96 7 E1159 E1162 10.1210/jc.2011-0254 21543425 PMC3135202

[B56] Blumsohn A Naylor KE Timm W Eagleton AC Hannon RA Eastell R Absence of marked seasonal change in bone turnover: a longitudinal and multicentre cross-sectional study J Bone Miner Res 2003 18 1274 1281 10.1359/jbmr.2003.18.7.1274 12854838

[B57] Woitge HW Scheidt-Nave C Kissling C Leidig-Bruckner G Meyer K Grauer A Scharla SH Ziegler R Seibel MJ Seasonal variation of biochemical indices of bone turnover: results of a population-based study J Clin Endocrinol Metab 1998 83 68 75 10.1210/jc.83.1.68 9435418

[B58] Nielsen HK Brixen K Bouillon R Mosekilde L Changes in biochemical markers of osteoblastic activity during the menstrual cycle J Clin Endocrinol Metab 1990 70 1431 1437 10.1210/jcem-70-5-1431 2110577

[B59] Chiu KM Ju J Mayes D Bacchetti P Weitz S Arnaud CD Changes in bone resorption during the menstrual cycle J Bone Miner Res 1999 14 4 609 615 10.1359/jbmr.1999.14.4.609 10234583

[B60] Black AJ Topping J Durham B Farquharson RG Fraser WD A detailed assessment of alterations in bone turnover, calcium homeostasis, and bone density in normal pregnancy J Bone Miner Res 2000 15 3 557 563 10750571 10.1359/jbmr.2000.15.3.557

[B61] Dorota D-K Bogdan KG Mieczyslaw G Bozena L-G Jan O The concentrations of markers of bone turnover in normal pregnancy and pre-eclampsia Hypertens Pregnancy 2012 31 166 176 10.3109/10641955.2010.484084 20822429

[B62] Naylor KE Iqbal P Fledelius C Fraser RB Eastell R The effect of pregnancy on bone density and turnover J Bone Miner Res 2000 15 129 137 10.1359/jbmr.2000.15.1.129 10646122

[B63] Hannon R Blumsohn A Naylor K Eastell R Response of biochemical markers of bone turnover to hormone replacement therapy: impact of biological variability J Bone Miner Res 1998 13 7 1124 33 10.1359/jbmr.1998.13.7.1124 9661076

[B64] van Staa TP Leufkens HG Cooper C The epidemiology of corticosteroid-induced osteoporosis: a meta-analysis Osteoporos Int 2002 13 777 787 10.1007/s001980200108 12378366

[B65] Schett G Osteoimmunology in rheumatic diseases Arthritis Res Ther 2009 11 1 210 10.1186/ar2571 19232069 PMC2688223

[B66] Wheater G Hogan VE Teng YKO Tekstra J Tuck SP Lafeber FP Huizinga TWJ Bijlsma JWJ Francis RM Datta HK van Laar JM Suppression of bone turnover by B-cell depletion in patients with rheumatoid arthritis Osteoporos Int 2011 12 3067 3072 21625887 10.1007/s00198-011-1607-0

[B67] Yamaguchi T Sugimoto T Bone metabolism and fracture risk in type 2 diabetes mellitus Endocr J 2011 58 8 613 624 10.1507/endocrj.EJ11-0063 21778617

[B68] Inque M Tanaka H Moriwake T Oka M Sekiguchi C Seino Y Altered biochemical markers of bone turnover in humans during 120 days of bed rest Bone 2000 26 3 281 286 10.1016/S8756-3282(99)00282-3 10710002

[B69] Veitch SW Findlay SC Hamer AJ Blumsohn A Eastell R Ingle BM Changes in bone mass and bone turnover following tibial shaft fracture Osteoporos Int 2006 17 364 372 10.1007/s00198-005-2025-y 16362144

[B70] Vesper H Cosman F Endres DB Garnero P Hoyle NR Kleerekoper MK Mallinak NJS Application of biochemical markers of bone turnover in the assessment and monitoring of bone diseases; approved guideline NCCLS document 2004 24 22 1 37

[B71] Garnero P Vergnaud P Hoyle N Evaluation of a fully automated serum assay for total N-terminal propeptide of type I collagen in postmenopausal osteoporosis Clin Chem 2008 54 1 188 196 17998267 10.1373/clinchem.2007.094953

[B72] Chubb SAP Measurement of C-terminal telopeptide of type I collagen (CTX) in serum Clin Biochem 2012 45 12 928 935 10.1016/j.clinbiochem.2012.03.035 22504058

[B73] Mora S Pitukcheewanont P Kaufman FR Nelson JC Gilsanz V Biochemical markers of bone turnover and the volume and the density of bone in children at different stages of sexual development J Bone Miner Res 1999 14 1664 1671 10.1359/jbmr.1999.14.10.1664 10491213

[B74] Eastell R Garnero P Audebert C Cahall DL Reference intervals of bone turnover markers in healthy premenopausal women: results from a cross-sectional European study Bone 2012 50 5 1141 1147 10.1016/j.bone.2012.02.003 22348982

[B75] Bieglmayer C Kudlacek S The bone marker plot: an innovative method to assess bone turnover in women Eur J Clin Invest 2009 39 230 238 10.1111/j.1365-2362.2009.02087.x 19260953

[B76] Seibel MJ Lang M Geilenkeuser W-J Interlaboratory variation of biochemical markers of bone turnover Clin Chem 2001 47 8 1443 1450 11468235

[B77] Schafer AL Vittinghoff E Ramachandran R Mahmoudi N Bauer DC Laboratory reproducibility of biochemical markers of bone turnover in clinical practice Osteoporos Int 2010 21 439 445 10.1007/s00198-009-0974-2 19506793 PMC2817087

[B78] Bauer D Krege J Lane N Leary E Libanati C Miller P Myers G Silverman S Vesper HW Lee D Payette M Randall S National bone health alliance bone turnover marker project: current practices and the need for US harmonization, standardization, and common reference ranges Osteoporos Int 2012 23 2425 2433 10.1007/s00198-012-2049-z 22797491 PMC4011662

[B79] Lee J Vasikaran S Current recommendations for laboratory testing and use of bone turnover markers in management of osteoporosis Ann Lab Med 2012 32 105 112 10.3343/alm.2012.32.2.105 22389876 PMC3289774

[B80] Steiger P Cummings SR Black DM Spencer NE Genant HK Age related decrements in bone mineral density in women over 65 J Bone Miner Res 1992 7 625 632 1414480 10.1002/jbmr.5650070606

[B81] Ross PD Knowlton W Rapid bone loss is associated with increased levels of biochemical markers J Bone Miner Res 1998 13 2 297 302 10.1359/jbmr.1998.13.2.297 9495524

[B82] Crane M Davis T Kaldale R Black C Davies R Devas V Williams W Relating increases in bone mineral density and fracture risk reduction with early suppression in biomarkers of bone turnover: a literature-based meta-analysis of bisphosphonates treatments J Bone Miner Res 2005 20 Suppl 1 S95

[B83] Delmas PD Eastell R Garnero P Seibel MJ Stepan J The use of biochemical markers of bone turnover in osteoporosis Committee of Scientific Advisors of the International Osteoporosis Foundation. Osteoporos Int 2000 11 Suppl 6 S2 S17 10.1007/s001980070002 11193237

[B84] Schuit SC van der Klift M Weel AE de Laet CE Burger H Seeman E Hofman A Uitterlinden AG van Leeuwen JP Pols HA Fracture incidence and association with bone mineral density in elderly men and women: The rotterdam study Bone 2004 34 195 202 10.1016/j.bone.2003.10.001 14751578

[B85] Osteoporosis: assessing the risk of fragility fracture: NICE Clinical Guideline 146 (August 2012) http://www.nice.org.uk/nicemedia/live/13857/60399/60399.pdf

[B86] Blank RD Malone DG Christian RC Vallarta-Ast NL Krueger DC Drezner MK Binkley NC Hansen KE Patient variables impact lumbar spine dual energy x-ray absorptiometry precision Osteoporos Int 2006 17 768 774 10.1007/s00198-005-0050-5 16435075

[B87] The international society for clinical densitometry official position 2007 http://www.iscd.org/official-positions/4th-iscd-position-development-conference-adult/

[B88] Jordan N Barry M Murphy E Comparative effects of antiresorptive agents on bone mineral density and bone turnover in postmenopausal women Clin Interv Aging 2006 1 4 377 387 10.2147/ciia.2006.1.4.377 18046915 PMC2699644

[B89] Watts NB Diab DL Long-term use of bispohosphonates in osteoporosis J Clin Endocrinol Metab 2010 95 4 1555 1565 10.1210/jc.2009-1947 20173017

[B90] Finkelstein JS Wyland JJ Lee H Neer RM Effects of teriparatide, alendronate, or both in women with postmenopausal osteoporosis J Clin Endocrinol Metab 2010 95 4 1838 1845 10.1210/jc.2009-1703 20164296 PMC2853981

[B91] Meier C Seibel MJ Kraenzlin ME Adler RA From Biochemical markers of bone turnover – clinical aspects Contemporary Endocrinology: Osteoporosis: Pathophysiology and Clinical Management 2010 131 155 Humana Press

